# Tendon Yaw (TY) Angle: Direct Measurement of the Quadriceps Vector Resolves the Rotational Enigma of Recurrent Patellar Instability

**DOI:** 10.3390/medicina62010049

**Published:** 2025-12-26

**Authors:** Dinko Nizić, Marko Šimunović, Mario Josipović, Josip Vlaić, Ivan Levaj, Jure Serdar, Irijana Rajković, Josip Ćurić, Zoran Sulje, Mislav Jelić

**Affiliations:** 1 Special Hospital Agram, Trnjanska cesta 108, 10000 Zagreb, Croatia; dinko.nizic@gmail.com; 2 Medikol Polyclinic, Voćarska cesta 106, 10000 Zagreb, Croatia; irijana.rajkovic@medikol.hr (I.R.);; 3 Department of Orthopaedic Surgery, University Hospital Center Zagreb, School of Medicine, University of Zagreb, Šalata 6–7, 10000 Zagreb, Croatia; ortopedija@kbc-zagreb.hr (M.J.);; 4 Division of Paediatric Orthopaedic Surgery, Children’s Hospital Zagreb, Ulica Vjekoslava Klaića 16, 10000 Zagreb, Croatia; jvlaic@yaho.co.uk

**Keywords:** quadriceps tendon, tendon yaw, patellar instability, patellofemoral joint, computed tomography, patellar tilt, trochlear dysplasia, vastus medialis obliquus, knee rotation, tibial rotation, femoral rotation, diagnostic accuracy, reproducibility of results

## Abstract

*Background and Objectives*: To validate a computed tomography (CT) measure of tendon yaw (TY) and determine its diagnostic specificity, precision, and clinical relevance in recurrent patellar instability (RPI) in comparison with standard imaging tests (SIT1, SIT2), femoral trochlear dysplasia (FTD), and vastus medialis obliquus (VMO) morphology. *Materials and Methods*: This retrospective cross-sectional study included 113 subjects (187 knees) examined using a standardized CT protocol for RPI following strict exclusion criteria. TY, SIT1, and SIT2 were measured using predefined axial landmarks. VMO cross-sectional area was assessed at three standardized levels. Diagnostic performance, measurement precision, and interrater agreement were evaluated. *Results*: TY significantly distinguished recurrent dislocators from nondislocators (*p* = 0.003) and was independent of age, sex, laterality, and femoral, tibial, or knee rotation (*p* ≥ 0.42). A threshold of ≥22° demonstrated high diagnostic specificity (92%; 95% CI, 85–97%), with a normal cutoff defined as ≤12°. Measurement precision was approximately 90%. SIT1 and SIT2 were influenced by femoral and knee rotation but not tibial rotation. All imaging tests were associated with FTD (*p* < 0.0001). No significant correlation was found between any imaging test and VMO area, although VMO was reduced in recurrent dislocators and in women. *Conclusions*: TY is a direct and highly specific CT measure of extensor mechanism yaw (*z*-axis rotation) that avoids indirect osseous and soft-tissue surrogates, supporting confirmatory diagnostic assessment and preoperative planning in RPI.

## 1. Introduction

Assessing rotation of the quadriceps tendon (QT) with standard imaging tests (SITs) is important in evaluating recurrent patellar instability (RPI) [1]. Lateral patellar tilt (LPT) reflects lateral inclination of the patella and serves as an indirect marker of rotational alignment of the QT in which the patella is embedded. LPT is one of the most difficult RPI factors to correct [2], and its relationship with femoral trochlear dysplasia (FTD) is well established [3]. A widespread belief also exists that atrophy of the vastus medialis obliquus (VMO) contributes to LPT and represents a form of quadriceps dysplasia that should be managed with physiotherapy [2,4]. However, no imaging study has directly validated rotational misalignment of the QT itself.

The main objective of this cross-sectional retrospective imaging study was to validate a new measure of QT rotational misalignment. We assessed its correlation with SITs, FTD, and VMO measurements in patients with RPI.

## 2. Materials and Methods

Following approval by the Ethics Committee of the University Hospital Center Zagreb (19 June 2017; Class 8.1-17/122-2, No. 02/21-AG), a cross-sectional retrospective imaging study was conducted at the Clinical Department of Diagnostic and Interventional Radiology.

Subjects who underwent CT imaging according to the standardized RPI protocol between January 2013 and May 2019 were identified. Scanning was performed on a 16-slice Siemens CT scanner (Siemens Medical Systems, Erlangen, Germany) with the patient positioned supine, legs extended, feet together, and the quadriceps muscles relaxed. Technical parameters were as follows: 40 to 51 mA, 130 kV, and a slice thickness of 2 mm. Images were acquired in both bone windows (width, 1500 HU; level, 450 HU) and soft-tissue windows (width, 450 HU; level, 45 HU).

Measurements were performed using Sectra Workstation IDS7 (version 23.1.12.4638, Linköping, Sweden) independently by two radiologists blinded to each other’s results; however, due to the retrospective study design based on existing clinical records, complete blinding to clinical status was not feasible, in accordance with CONSORT/STROBE reporting expectations for retrospective imaging studies.

All available clinical and radiological data from the hospital information system and the Picture Archiving and Communication System (PACS) were reviewed. Between January 2013 and May 2019, 383 knees (217 subjects) were imaged according to the RPI CT protocol. Exclusion criteria were as follows: 1. incomplete or unavailable medical records, 2. gonarthrosis or patellofemoral arthrosis, 3. previous knee surgery, 4. MRI-confirmed meniscal tear, 5. MRI-confirmed injury of the medial patellofemoral ligament (MPFL), cruciate, or collateral ligaments, 6. generalized joint laxity, 7. previous fracture or bipartite patella without evidence of prior trauma or dislocation, 8. chromosomal abnormalities, 9. ankylosing or rheumatoid arthritis, 10. spastic diparesis or unilateral hemiparesis, 11. angular knee deformity, 12. hip joint pathology, and 13. patellofemoral syndrome (PFS). After applying these criteria, 196 knees (104 subjects) were excluded, including 2 with incomplete CT scans and 194 meeting clinical exclusion criteria. The final study population comprised 113 subjects (187 knees). Subjects were categorized into two groups: the dislocated patellar group with documented ≥2 recurrent patellar dislocations in the medical record and the nondislocated patellar group with no record of dislocation.

**Figure 1:**
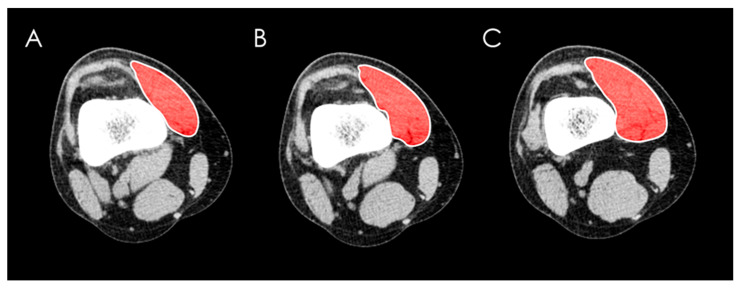
Measurement of the cross-sectional area of the vastus medialis obliquus (VMO) muscle by manually tracing its boundaries using the “area” tool on CT images at three levels: (**A**) the first ascending slice where the patella was no longer visible superiorly, (**B**) 1 cm above this slice, and (**C**) 2 cm above the first slice.

The cross-sectional area of the VMO muscle was measured by manually tracing muscular boundaries with a computer mouse using the “area” tool at three levels: 1. the first ascending slice where the patella was no longer visible superiorly, 2. 1 cm above this slice, and 3. 2 cm above the first slice (Figure 1).

**Figure 2:**
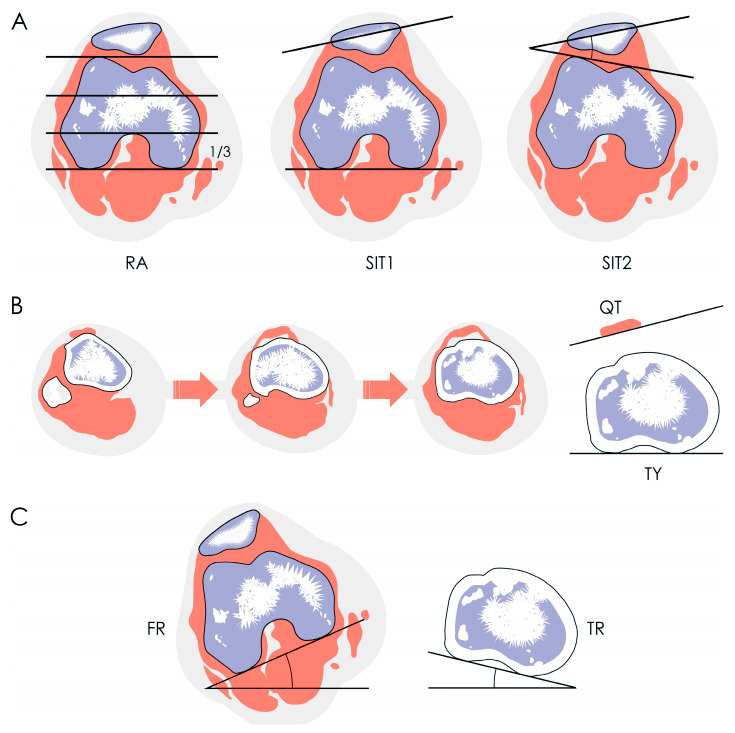
Schematic of measurements: measurement of lateral patellar tilt angles (**A**) on the axial CT image where the posterior intercondylar notch occupies approximately one-third of the anteroposterior femoral diameter (“Roman arch” slice) (**left**), according to the Fulkerson (**middle**) and Sasaki–Yagi (**right**) angles; tendon yaw angle on the first ascending CT image where the fibula was no longer visible (“headless cut”) (**B**); and rotational alignment of the femur (**left**) and tibia (**right**) (**C**). FR, femoral rotation (angle); QT, quadriceps tendon; RA, “Roman arch”; SIT1, standard imaging test 1; SIT2, standard imaging test 2; TR, tibial rotation (angle); TY, tendon yaw (angle).

**Figure 3:**
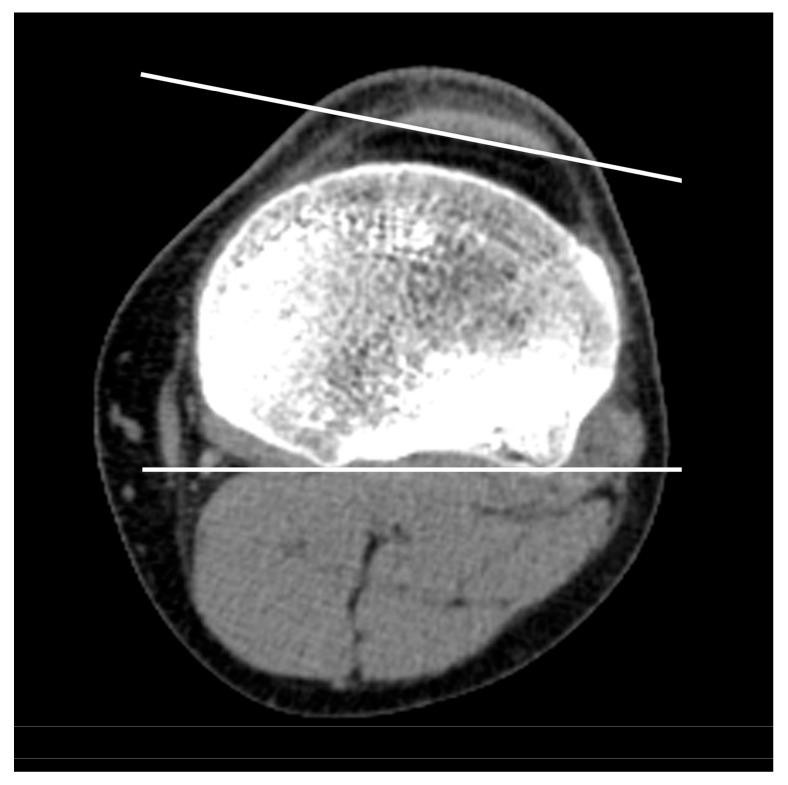
Tendon yaw (TY) angle measured on the first ascending CT image where the fibula was no longer visible (“headless cut”).

Patellar rotation was measured using the Fulkerson angle (SIT1), the Sasaki–Yagi angle (SIT2), and the new tendon yaw (TY) angle (Figure 2) where yaw is a biomechanical term indicating rotation around the *z*-axis. SIT1 was measured on the “Roman arch” slice (the axial CT image where the posterior intercondylar notch occupies approximately one-third of the anteroposterior femoral diameter) as the angle between the longest transverse patellar diameter and the posterior intercondylar line of the femur [5]. SIT2 was measured on the same slice as the angle between the longest transverse patellar diameter and the anterior intercondylar line of the femur [6]. The TY angle was measured on the first ascending CT image where the fibular head was no longer visible (hereafter referred to as the “headless cut”) as the angle between the posterior intertendinous line of the patellar tendon and the posterior intercondylar line of the tibia [7] (Figure 3). Unlike SIT1 and SIT2, TY directly quantifies *z*-axis rotational alignment of the tendon itself rather than the patella as its surrogate.

Rotational alignment of other structures was also measured. Femoral rotation (FR) was measured on the “Roman arch” slice as the angle between a line parallel to the CT table and the posterior intercondylar line of the femur [7,8]. Tibial rotation (TR) was measured on the “headless cut” as the angle between a line parallel to the CT table and the posterior intercondylar line of the tibia [7,8]. The difference between these two angles (FR minus TR) represented knee rotation (KR), calculated according to the modified Schneider–Tomczak method [7,8,9,10]. External rotation was assigned a positive value and internal rotation a negative value [7,8,10].

### Statistical Analysis

Normality was assessed with the Shapiro–Wilk test. Correlations were evaluated using Pearson and Spearman methods. Differences were tested using the paired-samples *t* test and the Wilcoxon test for paired samples (MedCalc, version 14.8.1; MedCalc Software, Ostend, Belgium). Power analysis targeted 90% statistical power at *p* < 0.05 to detect weak correlations (r = 0.30) in 112 knees and large paired differences (d = 0.50) in 44 and 47 knee pairs, respectively (G Power, version 3.1.9.2; Franz Faul, Kiel University, Kiel, Germany).

Accuracy was defined as agreement with the clinical reference standard, which is independent of imaging. Given that RPI is clinically overt, specificity (SP) was the primary accuracy metric in this setting.

Variability for TY was high relative to its mean across knees (standard deviation [SD], 5.7; mean, 13.6; coefficient of variation [CV], 42%), which complicates selection of normal cutoff values when using mean-based approaches. Given the high CV of the TY angle, the threshold was determined using the count ratio (CR) method rather than mean-based cut-offs, allowing direct calibration against the independent clinical diagnosis of RPI (which equals the clinical truth since the clinical diagnosis of patellar dislocation is straight-forward). To address this, for each averaged TY value a CR of total positives and total negatives was calculated according to clinical diagnosis of RPI. CR values below 1 were considered negative, CR values above 1 positive, and CR equal to 1 represented baseline. The test cutoff on the CR curve was defined as the lowest TY value followed by only positives. Sensitivity (SE), SP, and odds ratio (OR) with respective 95% confidence intervals (CI) were calculated.

Precision was defined as agreement of repeated measurements on a single knee. An SD from multiple measurements on a single knee was used as the estimate of measurement imprecision. Precision was defined as 100% minus CV%, where CV was calculated as SD divided by the arithmetic mean multiplied by 100% [11]. CV-based precision is appropriate for angular measurements because it normalizes variability across knees with different absolute angle magnitudes.

To be valid in the clinical setting, an imaging test required high accuracy and high precision, approximately 80% or higher. This threshold is commonly used in musculoskeletal imaging to indicate acceptable diagnostic performance.

Interrater agreement was assessed using the intraclass correlation coefficient (ICC), classified as moderate (0.41–0.60), substantial (0.61–0.80), and almost perfect (≥0.81) [12].

## 3. Results

All variables except TY and TR were non-normally distributed (Table 1).

SIT1 and SIT2 distinguished recurrent dislocators from nondislocators (*p* ≤ 0.002). They showed no association with sex (*p* ≥ 0.10), laterality (*p* ≥ 0.08), or TR (*p* ≥ 0.10), but were associated with FR (*p* = 0.02) and KR (*p* = 0.002). SIT2 decreased with age (r = −0.15; *p* = 0.035). In contrast, TY distinguished recurrent dislocators (t = 3.1; df = 89; *p* = 0.003) and showed no association with age (r = −0.08; *p* = 0.28), sex (z = 0.6; *p* = 0.54), laterality (t = −0.2; df = 92; *p* = 0.87), or any bone rotation measure (*p* ≥ 0.42) (Table 1).

**Figure 4:**
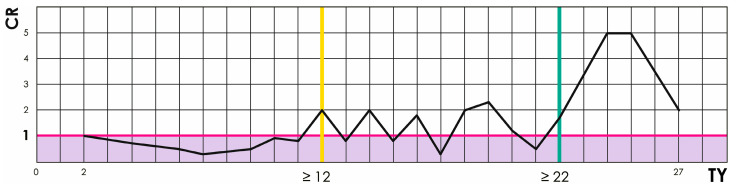
Count ratio (CR) curve of the tendon yaw (TY) angle showing physiological (12°, yellow vertical) and pathological (22°, green vertical) diagnostic cutoffs.

Variability of TY was high relative to its mean (SD, 5.7; mean, 13.6; CV, 42%). Nevertheless, its accuracy (defined as SP in this context) was 92% at a threshold of ≥22° (SE, 21% [13 to 30%]; SP, 92% [85 to 97%]; OR, 3.1 [95% CI, 1.2 to 7.7]; z = 2.4; *p* = 0.02), with the normal cutoff set at ≤12°. Precision was approximately 90% (Figure 4).

Interrater agreement for all imaging tests was almost perfect (ICC > 0.98).

All imaging tests were associated with FTD (*p* < 0.0001) and were mutually correlated (*p* < 0.0001), but none correlated with VMO (*p* ≥ 0.19). VMO was thinner in recurrent patellar dislocators (z = 2.7; *p* = 0.006) and in women (z = 4.4; *p* < 0.0001), and thickened with age (r = 0.18; *p* = 0.02), regardless of laterality (z = −0.7; *p* = 0.52) and FTD (z = −0.9; *p* = 0.35).

## 4. Discussion

The principal finding of this study is that, unlike SITs, the TY angle is both accurate and precise for quantifying QT yaw in RPI.

One should note that clinical terminology surrounding patellar instability is inconsistent in the available literature and is often applied interchangeably without clear definitions. “Patellofemoral instability” may refer to anything from subjective giving-way sensations to traumatic subluxation, and many authors do not specify whether objective dislocation is present. As a result, the intended meaning must frequently be inferred, sometimes incorrectly, and it is not always evident whether different authors are describing the same clinical entity. In contrast, the CT protocol used in this study was developed exclusively for RPI.

Most LPT methods, though (whether used routinely or not) rely on bony landmarks of the patella, trochlea, and femoral condyles. Osseous morphology is frequently altered in patients with RPI, which can distort LPT measurement [13]. Typical changes include condylar hypoplasia [14], varus or valgus deformity [15], and osteoarthritic remodeling with osteophytes. Wiberg’s classic classification is based precisely on variation in the medial and lateral patellar facets [16]. In clinical practice, the most common LPT techniques are those of Fulkerson [5,7,10] and Sasaki–Yagi [6,7,10], yet no consensus exists on pathological thresholds. Some authors consider up to 5° normal [17,18] and 20° pathological [19], while others suggest that angles above 10° warrant careful correlation with additional morphological indicators of RPI [17,20]. Beyond threshold ambiguity, traditional osseous reference points are affected by anatomical asymmetry and degenerative change [2,15]. Selecting the axial “Roman arch” slice is partly subjective, and joint effusion after acute dislocation can further inflate apparent LPT. Finally, LPT is associated with trochlear dysplasia in more than 77.7% of cases [17,21].

Whether VMO atrophy causes or follows increased LPT also remains debated. The prevailing view has been that LPT reflects VMO atrophy [1,19,22,23,24], but objective support is limited. Loss of full active extension has been linked to VMO insufficiency [25], inspiring conservative protocols focused on the terminal 15° of extension [25,26,27]. Several studies have questioned selective VMO strengthening in patellofemoral disorders [25,27,28,29]. Electromyography has generally failed to show selective VMO activation in terminal extension [30], and biomechanical cadaveric work reached similar conclusions [25].

Muscle force capacity scales with volume [31]. CT and MRI cross-sectional area measurements differ by only ~1.4%, supporting either modality for soft-tissue morphology [32]. Cross-sectional area correlates with strength [28] and can be measured reliably on multislice CT [32,33]. Still, MRI studies in RPI remain small [4,28,34]. Balcarek and Liu reported a non-significant trend toward smaller VMO area in recurrent dislocators versus controls (*p* = 0.07 and *p* = 0.16) in cohorts of ~30 per group [28,34]. A Wuhan group found greater VMO fiber angulation, higher LPT, and more frequent FTD in RPI [4]. Overall, robust evidence for a causal VMO role in RPI is lacking, and it remains unclear whether hypotrophy precedes dislocation or follows repeated redislocations and reduced activity [28].

Most available work has also centered on VMO morphology and LPT in patellofemoral pain syndrome (PFS) [23,35,36,37,38]. Functional MRI has not demonstrated meaningful activation differences among quadriceps heads in PFS [29]. PFS itself is a subjective, inconsistently applied label with broad differentials, including runner’s knee, anterior knee pain, lateral compression, plica, and osteochondritis dissecans [26,39]. It is commonly recorded as an isolated entity and may present with pain or instability without morphological signs of RPI [22].

Regarding conservative therapy, two approaches coexist: general quadriceps strengthening [26,30,40,41] and selective VMO training [18,23,35]. Syme showed both reduce pain in RPI, with no clear advantage of VMO-targeted regimens over general rehabilitation [27]. Ultrasound work by Giles demonstrated atrophy across all quadriceps components in PFS compared with controls [40].

Our results do not support a significant association between VMO and LPT by either imaging method. In a large sample, VMO atrophy appears more likely a consequence than a cause of RPI, challenging the traditional view [1,16,19,23,24,25,28,42]. Although thighs often look externally symmetrical, CT cross-sections at matched levels reveal a consistent reduction in quadriceps area on the symptomatic side [7]. Compensatory fat can mask this, creating a false impression of equal limb volume. Subclinical quadriceps hypotrophy reduces the effective extensor force and alters the quadriceps force tensor. The medial stabilizing moment falls, and a rotational component emerges within the extensor vector. The TY angle captures this functional direction of force rather than relying on morphological surrogates. In this context, the term “global quadriceps vector” denotes the single net resultant force generated by the combined action of all quadriceps heads and transmitted through the patella and tendon, rather than a distinct anatomical structure. The TY angle captures the value of this global quadriceps vector. LPT then becomes a systemic manifestation of reduced quadriceps integrity and asymmetric force distribution, not a local failure of VMO alone.

**Figure 5:**
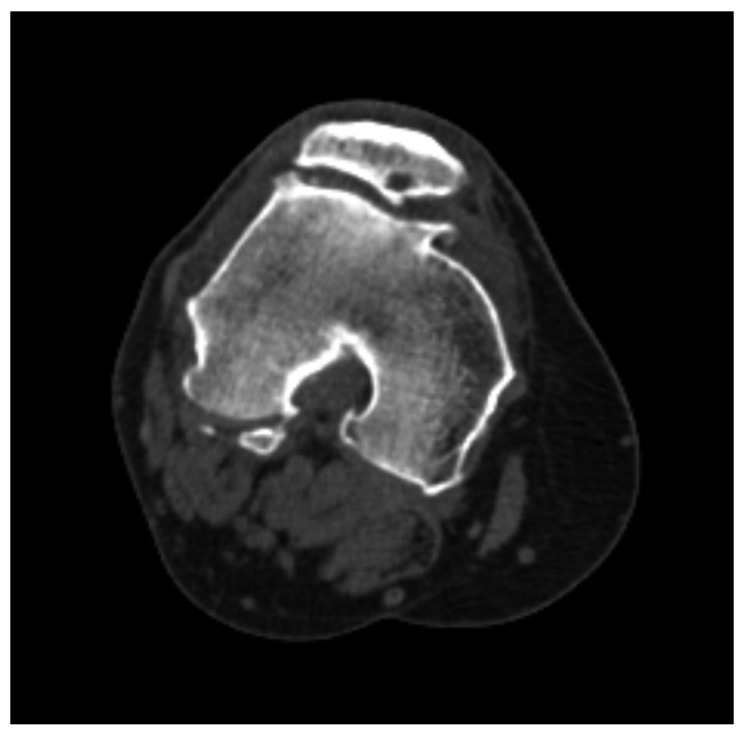
Axial CT image of the knee demonstrating advanced secondary osteoarthritic changes in the patellofemoral joint.

**Figure 6:**
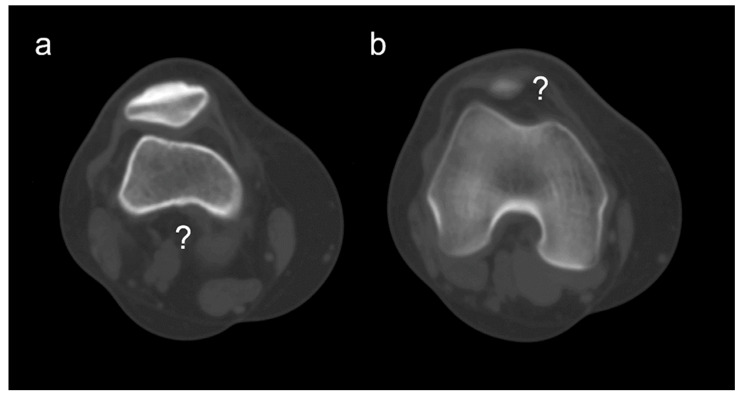
Axial CT images of the patellofemoral joint in two patients with patella alta. In the first patient, the posterior intercondylar notch cannot be visualized on the same axial slice as the patella (**a**), whereas in the second patient, the patella cannot be visualized on the same slice as the posterior intercondylar notch of the femur (**b**).

The construction of the TY angle avoids common landmark pitfalls—secondary osteoarthritic change (Figure 5) and patella alta or baja (Figure 6)—by using objective, reproducible references that are quick to identify in routine work. The tibial intercondylar midpoint reflects a key anatomical axis of the knee [43]. We set the reference level lower, at the apex of the fibular head, to avoid variability at the tibial plateau [8,44].

As with SIT1 and SIT2, the TY angle correlates with FTD. This is expected: the patella, as a sesamoid bone embedded in the extensor mechanism, glides within the femoral trochlea during flexion and extension. Alternative interpretations of this association would be physiologically implausible.

Standard patellofemoral metrics such as the tibial tubercle–trochlear groove (TT–TG) distance [19,45] conflate two different phenomena: translational displacement of the tibial tubercle and rotational deviation of the extensor vector. Separating them clarifies the pathomechanics. The tibial tubercle–tibial intercondylar midpoint (TT–TIM) distance quantifies translation—the linear offset between the tubercle and the tibial intercondylar midpoint regardless of whether measured on CT or MRI [7,8,46]—whereas TY quantifies rotation, the angular deviation of the quadriceps force vector relative to a tibial axis. Both are relatively independent of common confounders and can serve as pre- and postoperative gauges. Used together, they enable targeted correction of the dominant mechanism, translational or rotational, and provide a strong basis for future trials and potential therapeutic refinements in RPI. In essence, TT–TIM distance and TY angle may function as the “CRP of patellar instability”—objective, reproducible, mechanism-based markers that may uncover the true biomechanical disorder and permit accurate monitoring of therapeutic response. Nothing equivalent exists.

Finally, we propose a pathological threshold for TY-based LPT above 22°, a normal range up to 12°, and 12–22° as indeterminate, requiring clinical follow-up and correlation with other RPI markers.

This study has several limitations. The retrospective design is one, although it is unlikely that an event as painful and clinically overt as patellar dislocation would be missed in medical records. The cohort was derived from a single tertiary referral center, which may introduce a degree of institutional selection bias. Women constituted 70% of the cohort, reflecting both referral patterns and the known biomechanical predisposition related to pelvic width and stance angle that increase the lateral patellar vector. However, this sex imbalance should be considered when extrapolating the results. The cohort was predominantly Caucasian, and generalizability should therefore be interpreted primarily in the context of similar populations.

Although major potential confounding factors were rigorously controlled through strict a priori exclusion criteria rather than multivariable statistical modeling, residual unmeasured confounders cannot be entirely excluded in a retrospective design. In addition, external validation in independent cohorts was not performed and will be required before broader clinical generalization of the proposed TY thresholds. Finally, all measurements were CT-based, and while CT offers high geometric accuracy for rotational assessment, radiation exposure, albeit minimal, may limit its indiscriminate use in some clinical settings.

## 5. Conclusions

At length, TY is a direct and highly specific CT measure of extensor mechanism yaw (*z*-axis rotation) that avoids indirect osseous and soft-tissue surrogates, supporting confirmatory diagnostic assessment and preoperative planning in RPI.

## Figures and Tables

**Table 1:** Inter-variable correlations and differences.

Correlations			2–Tailed *p*	Differences		
Variables	r_p_	r_s_		t	z	Variables
.	.	.	<0.0001	.	4.4	VMO–S
TY–SIT1 *	.	0.51	<0.0001	.	.	.
TY–SIT2 *	.	0.51	<0.0001	.	.	.
TY–FTD *	.	0.31	<0.0001	.	.	.
SIT1–SIT2	.	0.90	<0.0001	.	.	.
SIT1–FTD	.	0.46	<0.0001	.	.	.
SIT2–FTD	.	0.44	<0.0001	.	.	.
.	.	.	0.001	.	–3.2	SIT2–RPI
.	.	.	0.002	.	3.1	SIT1–RPI
SIT1–KR	.	–0.22	0.002	.	.	.
SIT2–KR	.	–0.23	0.002	.	.	.
SIT2–FR	.	–0.22	0.002	.	.	.
.	.	.	0.003	3.1	.	* TY–RPI
SIT1–FR	.	–0.17	0.02	.	.	.
VMO–A	.	0.18	0.02	.	.	.
SIT1–A	.	–0.15	0.04	.	.	.
SIT2–A	.	–0.13	0.07	.	.	.
.	.	.	0.08	.	1.8	SIT1–L
.	.	.	0.10	.	–1.7	SIT1–S
SIT2–TR	.	–0.12	0.10	.	.	.
.	.	.	0.19	.	–1.3	SIT2–S
SIT1–VMO	.	–0.1	0.19	.	.	.
.	.	.	0.25	.	1.2	SIT2–L
TY–A *	.	–0.08	0.28	.	.	.
.	.	.	0.35	.	–0.9	VMO–FTD
SIT1–TR	.	–0.07	0.36	.	.	.
TY–KR *	.	0.06	0.42	.	.	.
.	.	.	0.52	.	–0.7	VMO–L
.	.	.	0.54	.	0.6	* TY–S
SIT2–VMO	.	–0.04	0.62	.	.	.
TY–VMO *	.	–0.03	0.66	.	.	.
TY–TR *	0.02	.	0.77	.	.	.
.	.	.	0.87	–0.2	.	* TY–L
TY–FR *	.	–0.01	0.94	.	.	.

* *TY* relation, *A* age, *FR* femoral rotation (angle), *FTD* femoral trochlear dysplasia, *KR* knee rotation (angle), *L* laterality, *RPI* recurrent patellar instability, *S* sex, *SIT1* standard imaging test 1, *SIT2* standard imaging test 2, *TR* tibial rotation (angle), *TY* tendon yaw (angle), *VMO* vastus medialis obliquus (muscle).

## Data Availability

Data are contained within the article.

## Abbreviations

A	age
CI	confidence interval (95%)
CR	count ratio
CRP	C-reactive protein
CV	coefficient of variation
df	degrees of freedom
FR	femoral rotation
FTD	femoral trochlear dysplasia
HU	Hounsfield units
ICC	intraclass correlation coefficient
KR	knee rotation
kV	kilovolts
L	laterality
LPT	lateral patellar tilt
mA	milliamperes
MPFL	medial patellofemoral ligament
No.	number
OR	odds ratio
PACS	Picture Archiving and Communication System
PFS	patellofemoral syndrome
QT	quadriceps tendon
RA	Roman arch
r	Pearson’s correlation coefficient
RPI	recurrent patellar instability (also known as patellofemoral instability)
r_s_	Spearman’s correlation coefficient
S	sex
SD	standard deviation
SE	sensitivity
SIT1	standard imaging test 1
SIT2	standard imaging test 2
SP	specificity
TR	tibial rotation
TT–TIM	tibial tuberosity–tibial intercondylar midpoint (distance)
TY	tendon yaw (angle)
VMO	vastus medialis obliquus (muscle)
